# Phytotoxicity of Alachlor, Bromacil and Diuron as single or mixed herbicides applied to wheat, melon, and molokhia

**DOI:** 10.1186/s40064-015-1148-7

**Published:** 2015-07-22

**Authors:** Yasser El-Nahhal, Nisreen Hamdona

**Affiliations:** Department of Environment and Earth Science, Faculty of Science, The Islamic University, Gaza, Palestine

**Keywords:** Alachlor, Diuron, Bromacil, Phytotoxicity, EC_50_, Synergistic, Antagonistic effect

## Abstract

This study investigated the phytotoxicity of herbicides applied singly or as mixtures to different crops under greenhouse conditions. Growth inhibition of the crops was taken as an indicator of phytotoxicity. Phytotoxicity of mixtures was estimated by calculating EC_50_ value in toxic units. EC_50_ (mg/kg soil) of Alachlor, Bromacil and/or Diuron were: 11.37, 4.77, 1.64, respectively, on *melon*; 0.11, 0.08, 0.24, respectively, on *molokhia*, and 3.91, 3.08, 1.83, respectively, on *wheat*. EC_50_ values of binary mixture tests of (Alachlor + Bromacil), (Alachlor + Diuron), and (Bromacil + Diuron) were 12.21, 5.84, 10.22 on *melon*, 0.982, 925.4, 38.1 on *molokhia*, and 0.673, 1.34, 0.644 on *wheat*. Tertiary mixture tests showed EC_50_ values (TU/kg soil) of (Alachlor + Bromacil + Diuron) was 633.9 on *melon*, 3.02 on *molokhia* and 32.174 on *wheat*. Diuron was more toxic than Alachlor and Bromacil to the tested crops based on individual tests. *Molokhia* was the most sensitive crop to herbicides. Binary mixtures showed a synergistic effect as compared to the tertiary mixtures.

## Background

Alachlor, Bromacil and Diuron are herbicides widely used for weed control all over the world. Alachlor is a chloroacetanilide herbicide, used to control annual grasses and certain broadleaf weeds in fields of corn, soybeans and peanuts. It inhibits protein synthesis in plant roots (Walker and Lawrence 1992). Bromacil belongs to Uracil family of herbicides used for brush control on non-cropland areas. It is especially useful against perennial grasses and used for selective weed control in pineapple and citrus crops (Redondo 1997). Diuron, one of the most commonly used herbicides, belongs to Urea derivatives that are applied in pre-emergence and post-emergence to control broadleaf weeds in a wide variety of annual and perennial broadleaf and grass weeds (Field et al. 1997; Gooddy et al. 2002). It has been classified as a slightly hazardous pesticide by WHO (USEPA 1994; Malato et al. 2002). Diuron is relatively persistent in the environment with a half-life of over 300 days.

The above-mentioned herbicides may enter freshwater ecosystems by spray drift, leaching, run-off, or accidental spills and present potential risks for several aquatic organisms. Moreover, the application of pesticides has resulted in contamination of food samples and agricultural commodities in many countries in the Middle East (El-Nahhal 2004). This situation may be associated with health disabilities (Abu Mourad 2000) and chronic diseases (Safi et al. 1993; Safi 2002). Furthermore, in spite of a relatively low solubility of the above-mentioned herbicides in water and the low vapor pressure, their application may have led to the contamination of groundwater in the USA, Canada, Europe, and the Middle East (Ritter et al. 1996; Riparbelli et al. 1986; Thurman et al. 1996). These contaminations may result in health risks and ecotoxicity. Moreover, application of the above-mentioned herbicides created soil and water contamination (El-Nahhal and Safi 2005).

Researchers have tried hard to develop less hazardous and environmentally safe formulations (El-Nahhal et al. 2001; Lagaly 2001; Nir et al. 2000; Rytwo 2005), including adsorption methods.

Moreover, application of herbicides in soil may result in their adsorption on clay minerals (Majka and Lavy 1977; Franco et al. 1997), soil organic matter (Grover 1975; Sánchez-Camazano et al. 2000), and organoclay complexes (El-Nahhal et al. 1998, 1999). This process may enhance the accumulation of herbicide concentrations in the topsoil and may endanger crops in the next growing season, or generate weed resistant genotypes that complicates the weed control process and makes control more costly (Thurman et al. 1996). Moreover, increasing herbicide concentration in the topsoil may damage or change the community structure of the cyanobacterial mats in the soil. The work of Abed et al. (2002), Awad et al. (2012), and recent results of Safi et al. (2014) support this suggestion.

In addition, the applied herbicide formulations contain active and inert ingredients, their toxic effects may differ from a single compound, and they may undergo synergistic or antagonistic effects that can alter the balance of an ecosystem.

### Toxicity to aquatic organisms

Several studies reported direct toxic effects on populations of phytoplankton and on green algae on surface water (Ma et al. 2003; Ma et al. 2002), reduced photosynthetic efficiency in corals of inshore reefs in the Great Barrier Reef (Shaw et al. 2012), and community-level effects (Lydy et al. 2004; Gilliom et al. 2006; Relyea 2006).

### Wheat, Jew’s mallow and melon in Palestine

*Whea*t is a winter season crop that depends on rain-fed farming whereas Jew’s mallow (*Corchorus olitorius *L.) and *melon* are essential economic, summer crops, that needs large quantities of water during the growing season (Ministry of Agriculture Palestine 2012). They are cultivated in different lands pre-treated with various herbicides. Very little information about the phytotoxicity of herbicide mixtures to crops is available elsewhere whereas in Palestine no reports are available in the field of mixture toxicity. Accordingly, this study was designed to: (1) characterize the phytotoxicity of Alachlor, Bromacil and Diuron as individual, binary and tertiary mixtures to *wheat,melon*, and *molokhia*, (2) study the responses of the three tested plants to herbicides concentration mixture, and (3) characterize the synergistic or antagonistic effects of these herbicides.

## Methods

**Figure 1:**
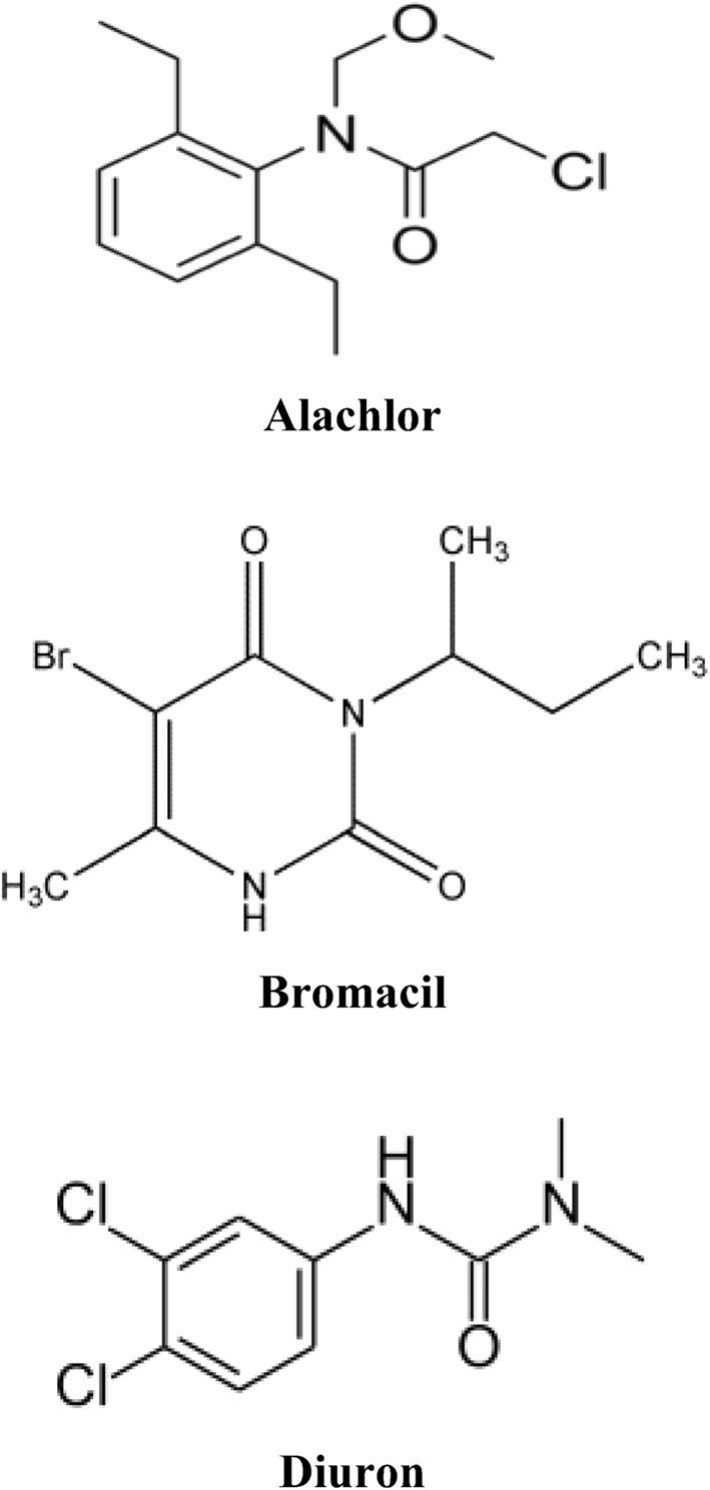
Chemical structure of the tested herbicides.

Alachlor, Bromacil and Diuron technical material, purity 99%, were purchased from the Sigma Chemical Co., Germany, and used as standard materials in this study. Some physic-chemical properties of these materials are presented in Table 1 and their chemical structures are shown in Figure 1. *Molokhia, melon* and *wheat* seeds were purchased from a local certified shop for agricultural products. Plastic pots were also purchased from a local certified shop for agricultural products in the Gaza Strip.

**Table 1:** Some physicochemical properties of the tested herbicides

Tested herbicide	K_ow_ log P	Solubility in water (mg/L)	Applied rate (Kg/ha)	Experimental rate^a^ (Mg/kg soil)
Alachlor	3.09	170.3	2	0.88
Bromacil	1.88	807	2	0.88
Diuron	2.85	36.4	0.6	0.25

Adopted from Tomlin (2000), ^a^ based on calculations by the authors.

### Soil collection

Soil samples were collected from areas believed to be free of herbicides with at least a 5-year history free from herbicide application. Soil samples were air-dried, sieved through a 2 mm mesh and stored in plastic bags under laboratory conditions. Soil pH, salinity, organic matter content and soil texture were analyzed according to the standard methods (Abadsa 2012).

### Preparation of herbicide stock solution

An amount of each herbicide below and above the recommended applied rate were mixed in soils and transferred to plastic pots for phytotoxicity tests. In addition, the concentrations in Table 2 were prepared and tested.

**Table 2:** Concentrations of herbicides (mg/kg soil) in single binary and tertiary mixture tests

Item	Single test			Binary mixture			Tertiary mixture
	Al	Br	Di	Al + Br	Al + Di	Br + Di	Al + Br + Di
C0	00	00	00	00	00	00	00
C1	0.06	0.06	0.005	0.03 + 0.03	0.03 + 0.003	0.03 + 0.003	0.02 + 0.02 + 0.001
C2	0.11	0.11	0.01	0.05 + 0.05	0.05 + 0.005	0.05 + 0.005	0.04 + 0.04 + 0.003
C3	0.22	0.22	0.02	0.11 + 0.11	0.11 + 0.01	0.11 + 0.01	0.07 + 0.07 + 0.006
C4	0.44	0.44	0.075	0.22 + 0.22	0.22 + 0.037	0.22 + 0.037	0.15 + 0.15 + 0.025
C5	0.88	0.88	0.1	0.44 + 0.44	0.44 + 0.05	0.44 + 0.05	0.29 + 0.29 + 0.033
C6	1.76	1.76	0.15	0.88 + 0.88	0.88 + 0.075	0.88 + 0.075	0.59 + 0.59 + 0.05

*Al* Alachlor, *Br* Bromacil, *Di* Diuron.

### Individual phytotoxicity tests

Phytotoxicity of Alachlor, Bromacil or Diuron to *wheat, melon*, and *molokhia* was assessed as growth inhibition. In this test, a technical amount of each herbicide (Alachlor, Bromacil and Diuron) below the solubility limit of each herbicide was dissolved in distilled water and used as a stock solution to prepare the required concentration of each herbicide as mentioned in Table 2. A wide range of concentrations of herbicide were prepared and examined to find an appropriate range of toxicity (linear concentration response relationship).

Following the procedure described previously (El-Nahhal 2003) the phytotoxicity tests were carried out with test plants in plastic pots under laboratory conditions. The required amounts of the herbicides were taken from the stock solution and added to each plastic pot. Then the soil was mixed thoroughly in plastic bags to insure an homogenized herbicide distribution and the soil was transferred back to the plastic pots. Ten seeds of each crop were sown in each pot, irrigated with 30 ml of fresh water and kept in the laboratory for 2 days. Subsequently they were irrigated with 20 ml each day or whenever necessary. Plant height/fresh weight were taken 2 weeks after germination and used as a parameter to measure growth inhibition (%GI) according to Eq (1) (El-Nahhal et al. 1998) and recent observations (El-Nahhal et al. 2013) taken as indicators of phytotoxicity.

1 $$\% {\text{ Growth inhibition}} = 100\; \times \;\left( {{\text{L}}_{0} - {\text{L}}_{\text{t}} } \right)/{\text{L}}_{0}$$

where, L_0_ and L_t_ are the plant length in the control and the treatment at each measured concentration.

The %GI values were regressed with the tested concentration to calculate the LC50, the concentration required to inhibit 50% plant growth.

### Phytotoxicity of mixtures

#### Binary mixture toxicity

Binary mixture toxicity of Alachlor, Bromacil and Diuron, which represent different chemical classes, were mixed together according to Table 2. The collected concentrations were then mixed together in a plastic bag to insure an homogenized mixture and the soil was transferred back to the pot experiment. Following the procedure mentioned above, phytotoxicity of the mixtures was then determined. The following mixtures MX1 = (Bromacil + Diuron), MX2 = (Alachlor + Bromacil) and MX3 = (Alachlor + Diuron) were prepared and tested.

A plot of % growth data versus the concentration of the herbicide was analyzed by linear regression to calculate the EC_50_.

#### Tertiary mixture toxicity

The concentrations mentioned in Table 2 were collected from the corresponding stock solution and mixed together to form a concentration of the tertiary mixture of Alachlor + Bromacil + Diuron. Then the concentration was mixed in soil as mentioned above and used for phytotoxicity tests. A plot of % growth data versus the concentration of the herbicide was analyzed by linear regression to calculate the EC_50_.

#### Calculation of phytotoxicity

According to El-Nahhal et al. (El-Nahhal et al. 1998), the % growth inhibition (%GI) represents phytotoxicity which is calculated according to Eq (1). The phytotoxicity values were regressed versus concentration, then converted to a log scale where necessary to calculate the LC50. Comparing LC50 values of the single phytotoxicity test indicates the phytotoxicity of the herbicide or the sensitivity of the test plant. For the binary or tertiary mixtures, the phytotoxicity of the mixtures was compared using toxic units (TU_S_). According to Sprogue and Ramsay (Sprague and Ramsay 1965), toxic units were calculated as:

$${\text{Toxic units}} = {\text{actual concentration in solution}}/{\text{lethal threshold concentration}}$$

Ishaque et al. (2006) defined toxic units as the concentration of a chemical in the toxic mixture divided by its single toxic concentration for the endpoint measured. To estimate the synergistic and/or antagonistic effects of herbicides mixtures, we calculated a mixture toxicity index (MTI) according to (Konemann 1981; Hermens et al. 1985).

MTI = 1 − (Log M/Log n), where M = ∑ c/EC50 at 50% effect in the mixture, and n = total number of compounds in the mixture and c is the concentration in mg/L. Accordingly, phytotoxicity of a mixture may be classified as an antagonistic effect if the MTI value is ≤0; partial addition if 0 < MTI < 1, and as a synergistic effect if the MTI ≥ 1.

All samples were kept in the same conditions described above. The 0.0 concentration always refers to the control sample. EC_50_ was estimated by the as described in the bioassay test (El-Nahhal et al. 1998; Bonnet et al. 2007).

### Statistical analysis

All experiments were performed in three replicates. Averages and standard deviations of growth inhibition were calculated and fitted to a regression analysis. The averages of growth inhibition were compared by Tukey’s test and P-values were determined to evaluate the differences among treatments.

## Results and discussion

### Phytotoxicity of a single herbicide test

**Figure 2:**
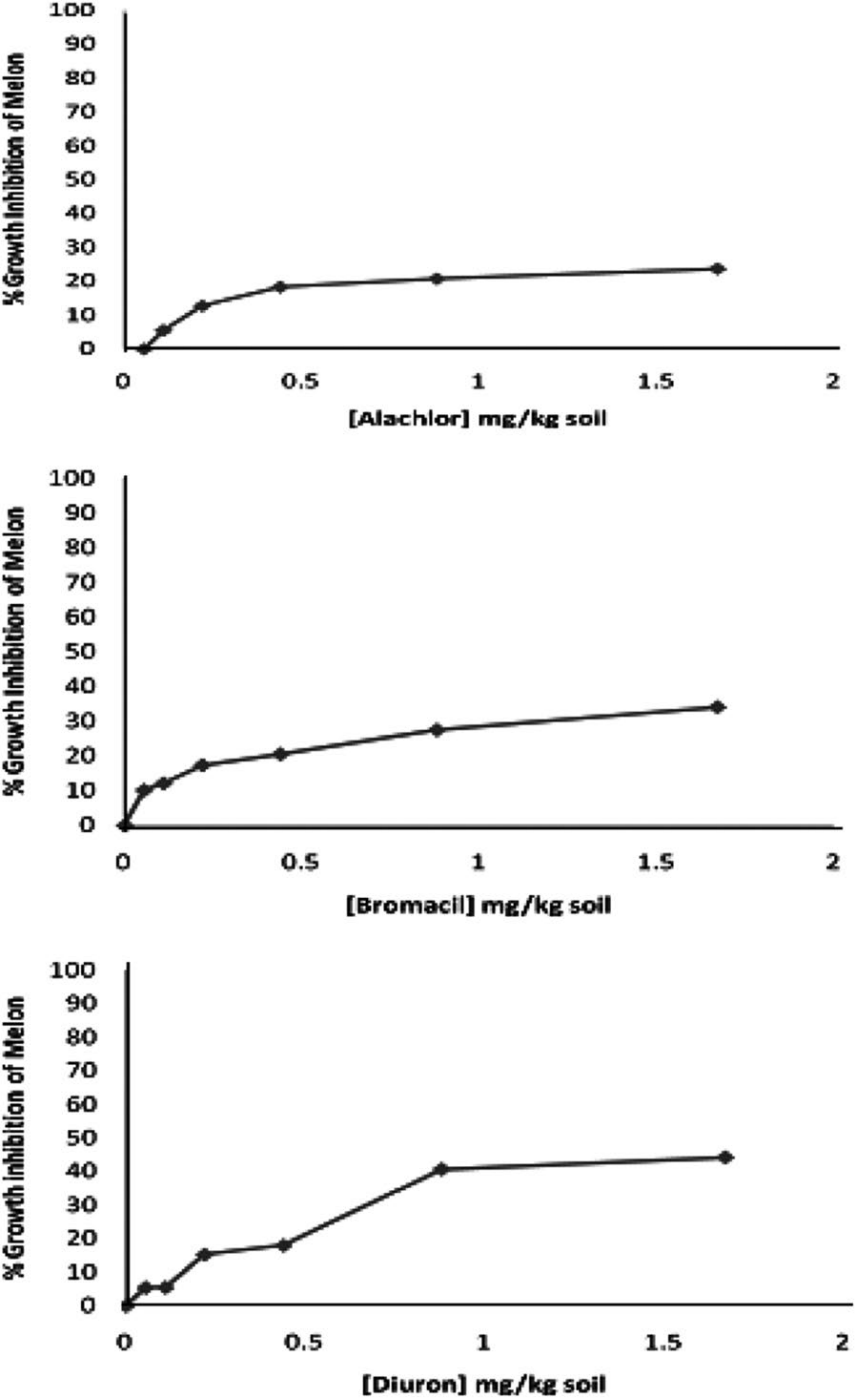
Effect of Alachlor, Bromacil and Diuron as a single test on *melon* growth.

**Figure 3:**
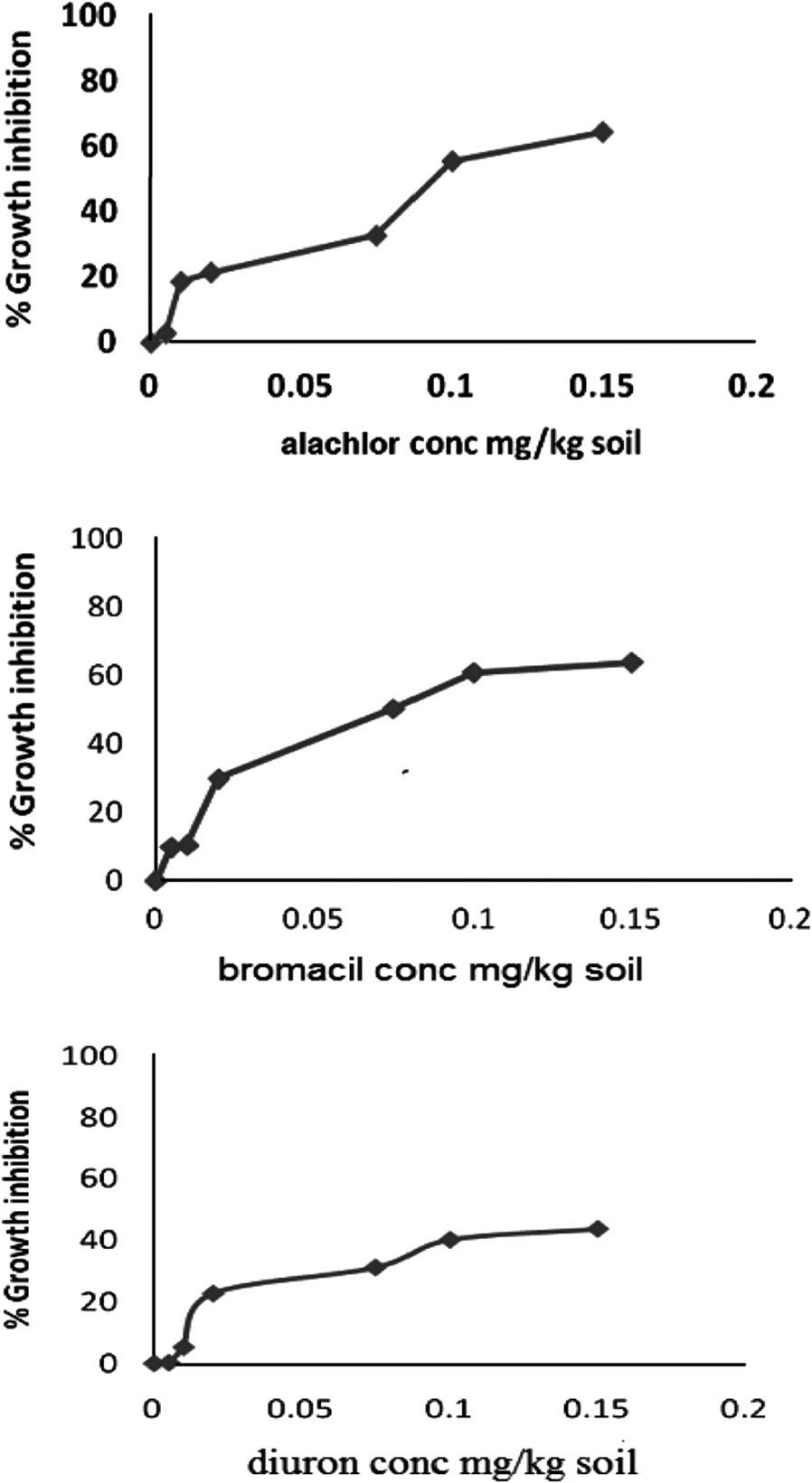
Effect of Alachlor, Bromacil and Diuron on *molokhia* growth.

**Figure 4:**
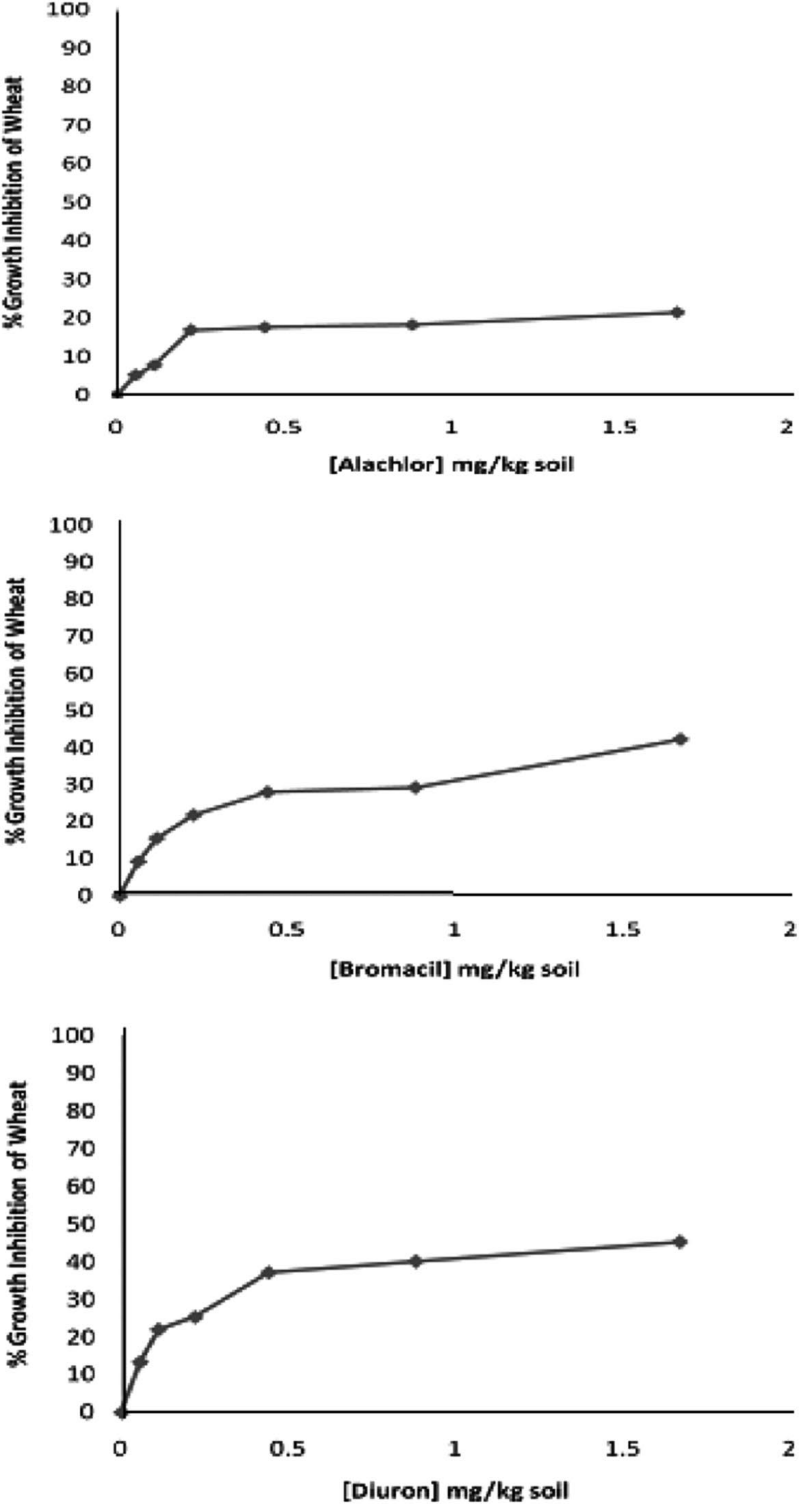
Effect of Alachlor, Bromacil and Diuron on *wheat* growth.

Phytotoxicity of Alachlor, Bromacil and Diuron as a single test on *melon, molokhia* and *wheat* are shown in Figures 2, 3 and 4, respectively.

The data presented in Figures 2, 3 and 4 clearly demonstrate that % growth inhibition (phytotoxicity) on *melon, molokhia* and *wheat* increased linearly with the concentration of Alachlor, Bromacil or Diuron increase in the soil, up to 0.5 mg/kg soil. Furthermore, above 0.5 mg/kg soil of each herbicide, a steady increase of growth inhibition was observed in all cases. However, % growth inhibition did not exceed 50% in *melon* and *wheat*, whereas it did in *molokhia*.

The explanation of these results is that at low concentrations of herbicide, the compounds are available in soil solution for plant uptake, accordingly considerable growth inhibition of the tested plant was observed. At high concentrations above 0.5 mg/kg soil the herbicides tend to distribute in the soil or may have leached down the root zone, consequently a reduction in % growth inhibition may be observed. This suggestion is supported by the results of (El-Nahhal et al. 1998, 1999) who found reductions of % growth inhibition due to leaching of herbicide concentration at deeper soil depths.

Furthermore, it has been shown that these plants may be resistant to the tested herbicides due to the enzyme system. Our results agree with Liu et al. (2013) who found that wheat along with winter annual or biennial weed of wheat in China were resistant to several acetolactate synthase (ALS) inhibitors.

In addition, El-Nahhal et al. (1998) found different effects of Alachlor on different test plants. Recently, Awad (2012) and Abadas (2012) found different % growth inhibition to the same herbicide. Moreover, Alachlor may undergo biodegradation in soil systems due to the growth of cyanobacteria. This suggestion is supported by the recent results of El-Nahhal et al. (2013) who found that fast dissipation of Acetochlor (alachlor isomer) in soil occurs due to incubation with cyanobacterial mats at different concentrations and periods.

Moreover, to compare the phytotoxic effect of the three tested herbicides (Table 3), we calculated the EC_50_ value of each herbicide from the corresponding log scale of % growth inhibition data presented in Figures 2, 3 and 4.

**Table 3:** Phytotoxicity parameters of Alachlor, Bromacil and Diuron on watermelon, molokhia and wheat

Herbicide	EC_50_ mg/kg soil	Equation	R^2^
*Melon*			
Alachlor	11.37	y = 0.2798X + 1.4045	0.958
Bromacil	4.77	y = 0.3615X + 1.4538	0.993
Diuron	1.64	y = 0.6904X + 1.55	0.946
*Molokhia*			
Alachlor	0.11	y = 0.4598X + 2.1444	0.909
Bromacil	0.08	y = 0.6077X + 2.3673	0.939
Diuron	0.24	y = 0.3291X + 1.9066	0.947
*Wheat*			
Alachlor	3.91	y = 0.4282X + 1.2922	0.946
Bromacil	3.08	y = 0.3342X + 1.5355	0.953
Diuron	1.83	y = 0.3381X + 1.61	0.957

Comparing the EC_50_ values of the tested compound on melon, it is obvious that Diuron has the lowest value (1.64 mg/kg), which is the most phytotoxic one, whereas, Alachlor has a value of 11.37 mg/kg, i.e. nearly 7 times higher than Diuron and 2.4 times higher than Bromacil. These results indicated that Alachlor is the safest herbicide among the tested compounds on the tested crops. These variations are probably due to different modes of action of the tested herbicides since each of them represents a different chemical class, apart from the fact that the growth patterns of the test plants are also different.

Furthermore, the regression equations (Table 3) indicated a linear mode of interaction, and R^2^ values close to 1 indicated a strong positive association between growth inhibition data (y) and herbicide concentration in soil (x) in the presented equations.

For the case of *molokhia*, Bromacil had the lowest EC_50_ value (0.08 mg/kg) making it the most toxic one while Diuron had the highest EC_50_ value (0.24 mg/kg) indicating the least phytotoxicity. Alachlor is nearly half of the EC_50_ value of diuron. Nevertheless, regardless of these variations in EC_50_, the three herbicides are still very toxic to *molokhia* since the tested concentrations are far below the applied rate.

In the case of wheat, the EC_50_ values had the same trend as for *melon*. Diuron was the most toxic one to *wheat*, (EC_50_ = 1.83 mg/kg soil) and Alachlor was the safest (EC_50_ = 3.91 mg/kg soil). Regression equations and R^2^ values in Table 3 supporting our evaluation.

Nevertheless, by comparing EC_50_ values of *molokhia* with those of* melon* and *wheat*, it is apparent that EC_50_ values for *molokhia* were the lowest, indicating that *molokhia* is the most sensitive plant for the tested herbicides. The sensitivity of *molokhia* plants may derive from the fact that it has a shorter period of growth than *melon* or *wheat*, accordingly it may not be able to develop a resistant genotype for herbicides. Moreover, Awad (2012) found that *molokhia* seeds are sensitive test plants for Diuron and Acetochlor herbicides in soil and can be used as a good soil pollution indicator. In addition, El-Nahhal et al. (2013) revealed that Diuron was highly adsorbed in the soil profile and was available for plant uptake during the growth season, thus a more phototoxic effect to the test plant was found. In addition, Safi et al. (2014) reported that Diuron was more resistant to biodegradation in soil and water systems, thus it was very toxic to the test plants. The regression values (R^2^) of the linear relationships of all tested compounds ranged from 0.909 to 0.993, indicating strong positive associations between % growth inhibition (y) and herbicide concentration (x) in all cases of single toxicity tests. These results agree with a previous report (Chen et al. 2003) that found a similar trend for other cases. Moreover, the variation of EC_50_ values of the tested compounds may also be explained by two factors; the value of K_OW_ of each herbicide (Table 1) and because Diuron has a high adsorption coefficient in soil (El-Nahhal et al. 2013) that enables Diuron to remain longer in the top soil.

Statistical analysis of the effects of the tested herbicides on different plants showed that the effects of Alachlor and Diuron on *molokhia* and *wheat* are nearly similar. The *p* value ranged between 0.09 and 0.1 indicating no significant difference, whereas the effects on *wheat* had significantly different p-values less than 0.01. By comparing the effects of Alachlor and Bromacil on wheat, one can conclude that both herbicides have similar effects and no statistical differences were detected. The P-value was 0.13, whereas the effects on *molokhia* and melon had significantly different p-values of less than 0.01.

The effects of Bromacil and Diuron on the 3 test plants were significantly different, with p-values less than 0.01. These values agree with the presented results in Figures 2, 3 and 4.

### Phytotoxicity of mixtures

#### Binary mixtures

**Figure 5:**
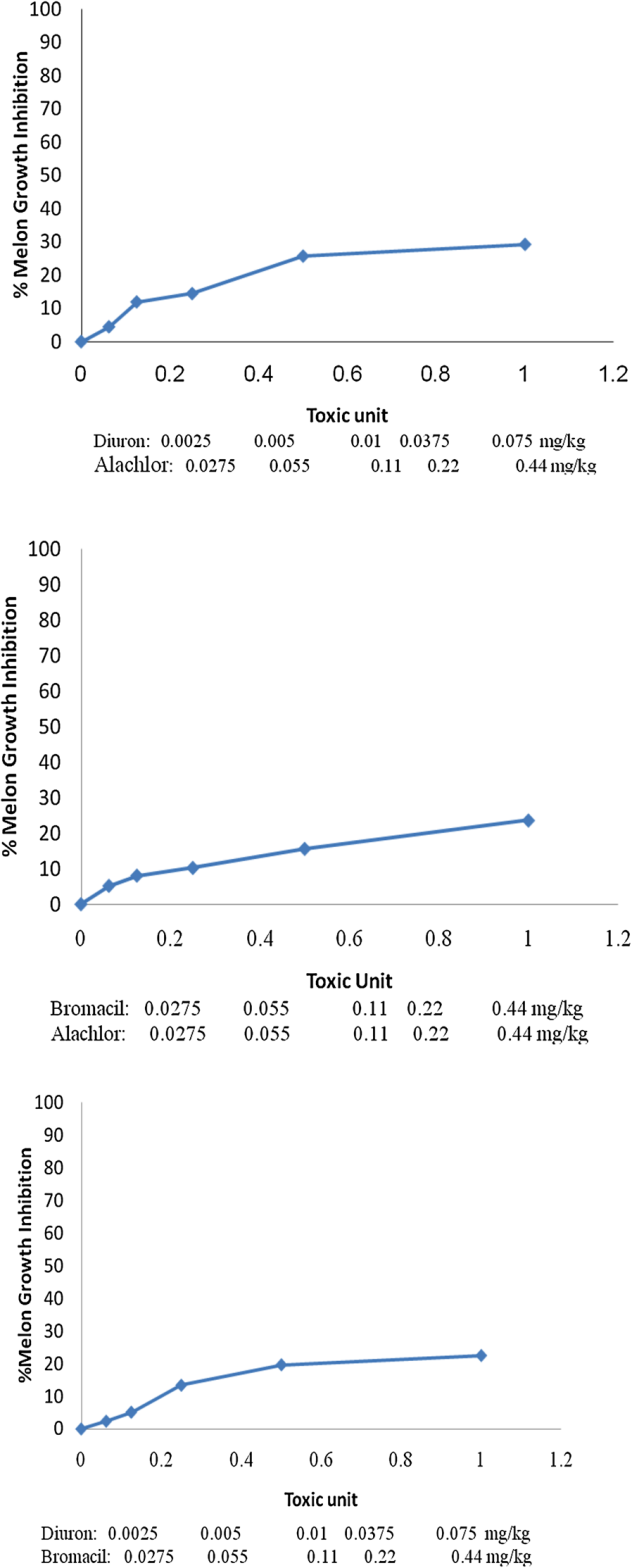
Effect of binary mixture on *melon* growth.

**Figure 6:**
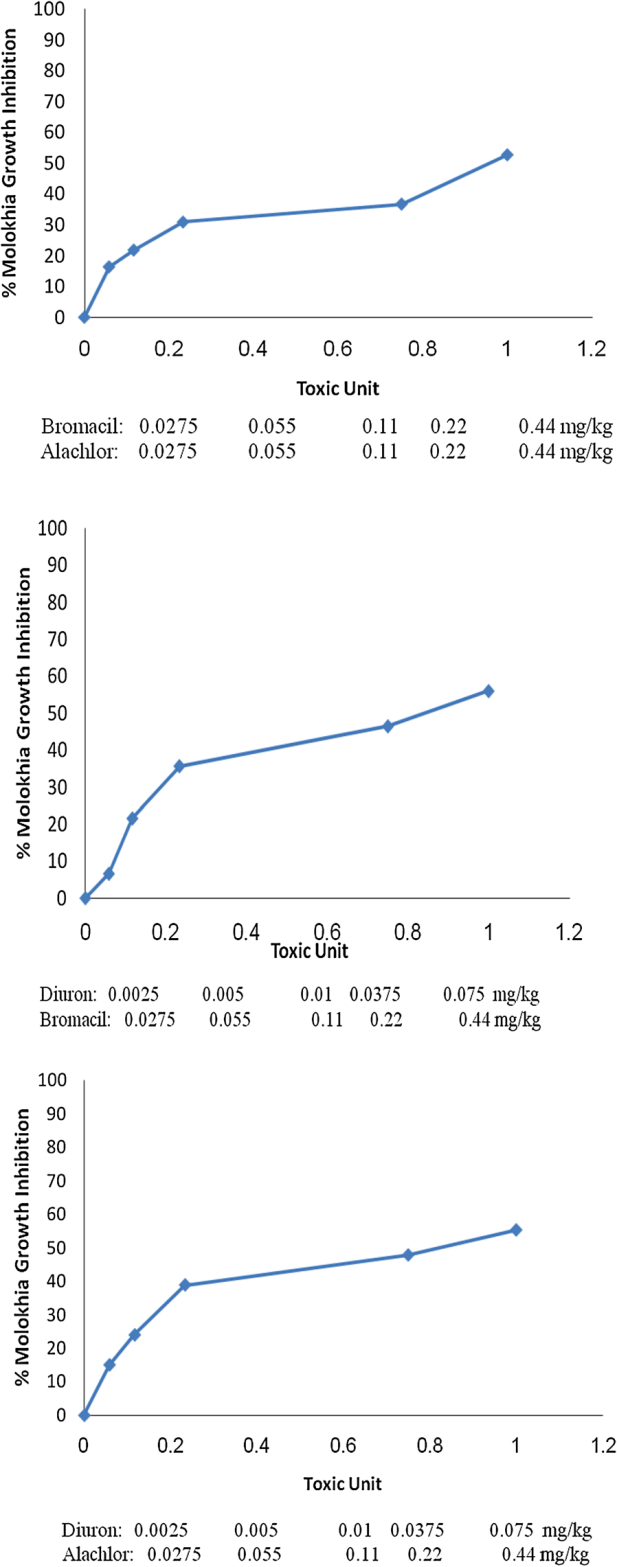
Effect of binary mixture on *molokhia* growth.

**Figure 7:**
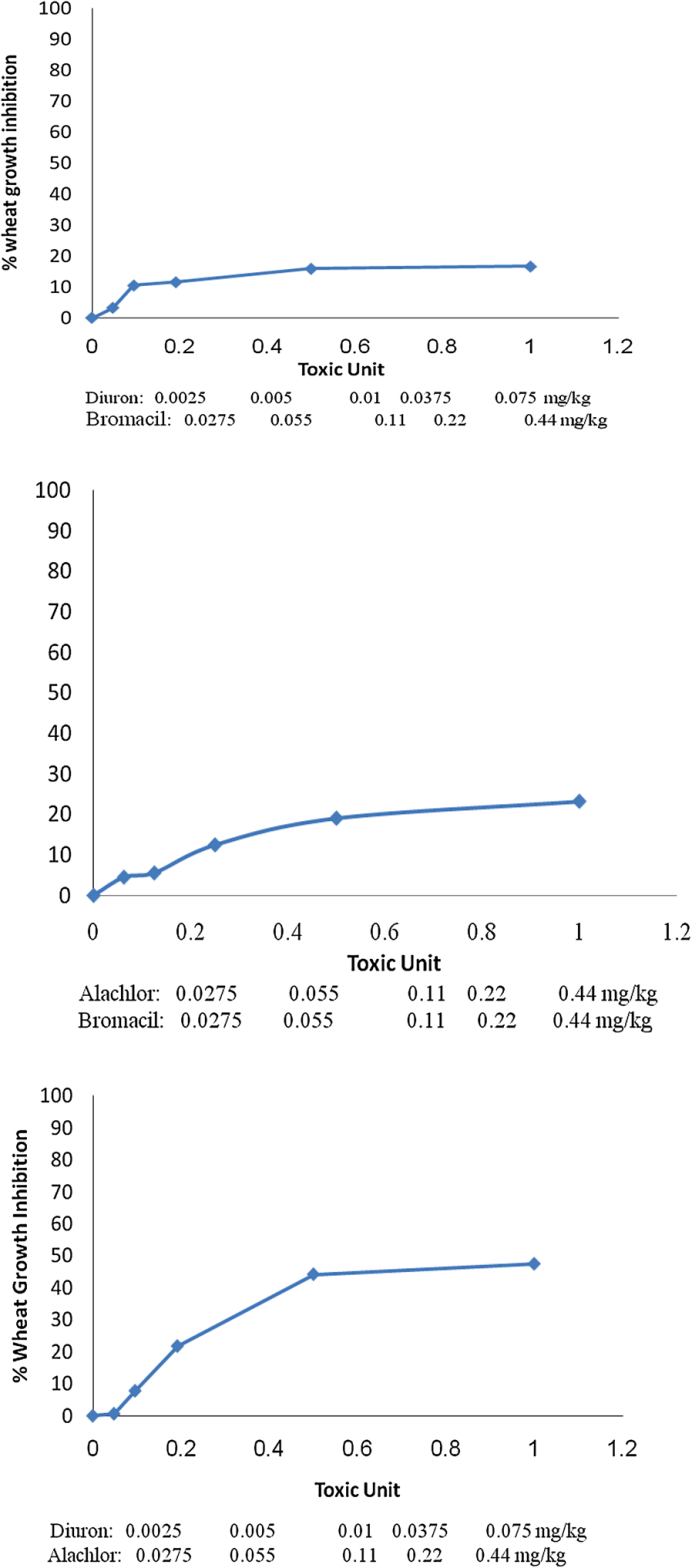
Effect of binary mixture on *wheat* growth.

Toxicity of binary mixtures of herbicides to the tested plants is shown in Figures 5, 6 and 7. The figures clearly demonstrate increased % growth inhibition as the concentration of the herbicide mixture increased in the soil. However, the toxicity tests have similar trends but different magnitudes of plant response. To evaluate quantitatively the phytotoxicity of binary mixtures, the EC_50_ values of mixtures were calculated and presented in Table 4. The EC_50_ values of the binary mixtures on melon (Table 4) clearly demonstrated that MX1 (Alachlor + Diuron) had the lowest EC_50_ of phytotoxicity and was the most toxic one (EC_50_ = 8.92 TU/kg soil), followed by an (Bromacil + Diuron) MX2 mixture (EC_50_ = 28.52 mg/kg soil), and (Alachlor + Bromacil) being the safest one among the mixtures (EC_50_ = 83.51 TU/kg soil). These results indicate that mixing Diuron with Alachlor or Bromacil produces high phytotoxicity that can be referred to as a partial synergistic effect. However, mixing Bromacil with Alachlor produced a high value of EC_50_ (83.51 TU/kg soil), which can be referred to as antagonistic effect. Our results agree with a recent study (Gatidou et al. 2015) that found a synergistic effect when mixing Diuron with linuron on duckweed control.

**Table 4:** Phytotoxicity parameters of binary and tertiary mixtures of Alachlor, Bromacil and Diuron on* melon, molokhia* and *wheat*

Mixture	EC_50_ (TU/kg soil)	Equation	R^2^
*Melon*			
Al + Di	8.92	y = 21.142X + 29.912	0.967
Al + Br	83.51	y = 14.819X + 21.522	0.933
Br +Di	83.51	y = 18.176X + 23.55	0.972
Al + Br + Di	11060.65	y = 8.771X + 14.532	0.93
*Molokhia*			
Al + Di	0.72	y = 31.288X + 54.512	0.975
Al + Br	1.35	y = 25.529X + 46.639	0.894
Br + Di	0.73	y = 37.062X + 55.003	0.973
Al + Br + Di	1.93	y = 21.221X + 43.927	0.77
*Wheat*			
Al + Di	0.982	y = 43.142 X + 50.33	0.955
Al + Br	38.1	y = 16.956X + 23.194	0.964
Br + Di	925.4	y = 10.751X + 18.109	0.902
Al + Br + Di	9	y = 19.996X + 30.918	0.93

For *molokhia*, mixing Diuron with Alachlor or Bromacil yielded a nearly similar phytotoxicity and can be referred to as a partial synergistic effect. The nearly high value of EC_50_ of Alachlor with Bromacil (1.35 TU kg/soil) suggests that an antagonistic effect occurs when mixing Alachlor and Bromacil. This indicates that mixtures containing Diuron are more toxic than mixtures without it.

The explanation is that the herbicide molecules in the mixture tests tend to interact with each other through hydrophobic interactions or π–π interactions (El-Nahhal et al. 2001) and may better interact with the active site in the test plant. Accordingly, synergism or a more phytotoxic effect was observed. This explanation agrees with a previous study (El-Nahhal and Safi 2004) that found organic molecules dissolve into each other and form a larger molecule that can react with clay mineral surfaces to produce an organo-clay complex able to maintain the slow release of the herbicide for the complex; accordingly a more toxic effect was observed. El-Nahhal and Lagaly (2005) found similar results with pesticide formulation. Furthermore, it may be suggested that mixing two herbicides together and adding them to soil enhances their adsorption to the clay or organic matter fraction in soil. Consequently, the herbicides molecules are retained in the topsoil layer and thus more herbicidal activity may be observed. This suggestion can be supported by the results of Undabeytia et al. (2008) who found synergism in adsorption of Alachlor and Atrazine when they were mixed together.

For the case of wheat, the trend is not similar. However, mixing Diuron with Alachlor produced a partially synergistic effect (EC_50_ = 0.982 TU/kg soil), whereas mixing Diuron with Bromacil produced an antagonistic effect (EC_50_ = 925.4 TU/kg soil). In contrast to the above cases, mixing Bromacil with Alachlor produced partial synergistic effects.

Our results agree with (Kerkez 2013) who found that Diuron and its mixtures were very toxic to cyanobacteria that have chlorophyll, a common phenomenon with higher plants.

The EC_50_ values of the binary mixtures on *wheat* (Table 4) clearly show that mixtures containing Alachlor were the most toxic ones and have the lowest TU. In contrast, mixtures that did not contain Alachlor had the highest EC_50,_ which in some cases were several hundred times higher. This indicates that Alachlor is responsible for the toxicity of the mixture against wheat. These results agree with (El-Nahhal et al. 1998, 2013a) who found that wheat was sensitive to Alachlor and Acetochlor, respectively. Statistical analysis of the phytotoxic effects of the mixtures on the test plant showed significant differences on melon growth p-values, which were less than 0.01. No significant differences were detected on the effect of mixtures on *molokhia* and *wheat*, indicating similar effects of p-values above 0.05 (data not shown). The explanation of these variations is given above.

#### Tertiary mixtures

**Figure 8:**
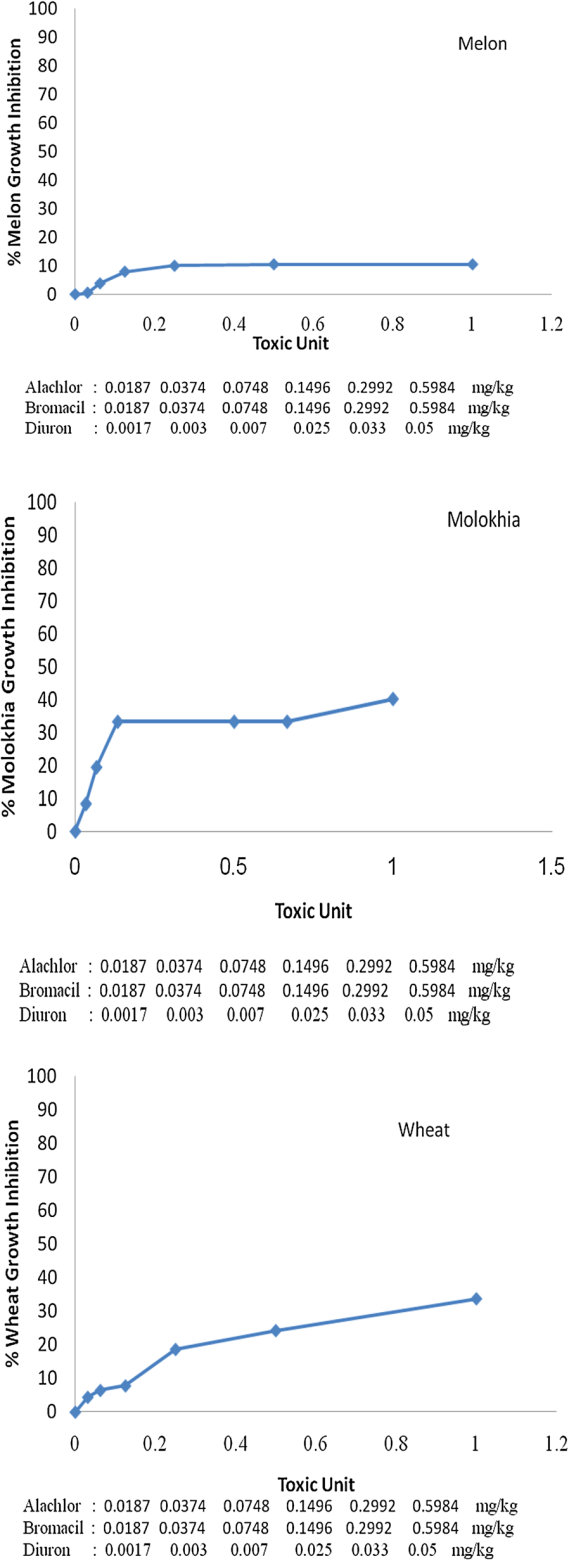
Effect of tertiary mixture on *melon*, *molokhia* and *wheat* growth.

Phytotoxicity of tertiary mixtures (Figure 8) clearly demonstrated that tertiary mixtures containing Alachlor and Diuron were less toxic to *wheat* and *molokhia* and less toxic to *melon*. These results suggest that melon can be a tolerant plant or can metabolize the toxic effects of herbicides to the least toxic ones. Furthermore, mixing the 3 herbicides together evenly may enhance the formation of one molecule of larger size that can penetrate the plant root and increase toxicity. This suggestion is supported by the results of (El-Nahhal and Safi 2004) who found that addition of phenanthrene molecules to water containing soluble organic molecules enhanced its solubility and that both molecules reacted together as one molecule on the clay surfaces. However, it may be hypothesized that the interaction in the tertiary mixture can be predicted from a knowledge of the binary interactions. Accordingly, the tertiary mixture of this study can be referred to as antagonistic effect. Our hypothesis agrees with Cedergreen et al. (2012) who predicted the tertiary mixture effects, based on using a stepwise modeling approach of incorporating the information held in binary mixtures into a tertiary mixture model.

Nevertheless, in comparing the EC_50_ values (Table 4), the value for molokhia (1.93 TU/kg) was the lowest of all, the value in wheat was 9 TU/kg soil, and the value on melon was the highest with 11060.65 TU/kg soil.

Comparing the effects of single, binary and/or tertiary mixtures of the tested herbicides showed variations in the EC50 values. Analysis of these data and calculations ofthe MTI (Table 5) showed negative values of binary and tertiary mixtures. These values indicate antagonistic effects. However, comparing the MTI values in melon showed extreme negative values in comparison with those for *molokhia or wheat.*

**Table 5:** Mixtures Toxicity Index (MTI) values of the mixtures

Crop	Mixture	MTI
*Melon*	Al + Br	−9.57
	Al + Di	−6.3
	Br + Di	−8.02
	Al + Br + Di	−6.96
*Molokhia*	Al + Br	−7.08
	Al + Di	−6.16
	Br + Di	−6.2
	Al + Br + Di	−1.33
*Wheat*	Al + Br	−8.43
	Al + Di	−3.12
	Br + Di	−13.04
	Al + Br + Di	−0.49

It may be possible to consider the MTI values of mixtures that have values close to zero as partially synergistic effects. Accordingly, the tertiary mixture (Al + Br + Di) on molokhia and wheat with MTI values equal to −1.33 and −0.49, and binary mixtures (Al + Di) in wheat with value equals to −3.12, are in the synergistic effects category. The mixtures that have MTI values less than −3.22 can be categorized as antagonistic mixtures. These results agree with the data presented in Figures 6, 7 and 8. It was proposed that mixing two or more herbicides together may result in additive, synergistic, and/or antagonistic effects. In the first case, the activity of the mixture is equal to the sum of the activities of all herbicides in the mixture when these herbicides are applied separately. In the second and third cases, however, the activity of the mixture is greater or lower, respectively, than the sum of the activities of all herbicides in the mixture (Hatzios and Penner 1985). In addition, we considered that a mixture has a synergistic effect if it caused greater growth inhibition to the test plant than the single component did for the same concentration. The mixture can be regarded as an antagonistic effect if the value of EC_50_ of the single toxicity test in mg/L is lower than its value in the mixture. Itis suggested that antagonistic effects of herbicides may emerge from physiological bases and interactions between the mixture and the test plant. This argument can be supported by the results of Ferreira et al. (1995), who revealed the physiological basis of antagonism among herbicides and plants.

## Conclusion

This study assessed variations of *melon, molokhia* and *wheat* responses to the herbicides Alachlor, Bromacil and Diuron. The single toxicity test indicated that *molokhia* was the most sensitive plant.

The EC_50_ value of single tests clearly demonstrated that Diuron is more toxic than Alachlor and Bromacil. Furthermore, phytotoxicity of binary mixtures indicted the highest toxicity of mixtures that contained Diuron.

The phytotoxicity of herbicides to *melon* and wheat follows the order Diuron > Bromacil > Alachlor, whereas the phytotoxicity in *molokhia* was Bromacil > Alachlor > Diuron.

Phytotoxicity of binary mixtures on *melon* and *molokhia* follows the order Al + Di > Br + Di > Al + Br, whereas phytotoxicity on *wheat* was Al + Di > Al + Br > Br + Di.

Phytotoxicity of tertiary mixtures in plants was *molokhia* >* wheat* > *melon*. The antagonistic effect was shown in all mixtures due to the negative values of MTI but mixtures that had MTI values close to zero were rated as partially synergistic regardless of the negative value of MTI. It is recommended that the application of combinations of herbicides should avoid diuron in mixtures. Moreover, application of herbicides for weed control should take into consideration the history of herbicide application to a given field. Application of combinations of herbicides should consider plant rotation.
